# Researches on Multiple Abscesses, and on the Morbid Effects of the Presence of Pus in the Vascular System

**Published:** 1843-01

**Authors:** 


### ART. XI.

*Recherches sur les Abcès Multiples, et sur les Accidents qu'amene la présence du Pus dans la Systême Vasculaire.* Thèse par FELIX D'ARCET, M.D.—*Paris,* 1842. 4to, pp. 85.

*Researches on Multiple Abscesses, and on the Morbid Effects of the presence of Pus in the Vascular System.* By F. D'ARCET, M.D.—*Paris,* 1842.

IT is known to all engaged in the practice of surgery that purulent deposits in distant parts, sero-purulent effusions into the serous and synovial cavities, and typhoid symptoms of the worst character are of frequent occurrence after wounds and operations, and that the same events not rarely supervene upon some local diseases not connected in their origin with external injury. This subject has appeared to the author of this thesis to admit of further elucidation by experiment; for, however fully these effects have been described, however attentively the circumstances under which they have taken place have been observed, however fully the symptoms accompanying them have been noted, there is as yet no agreement of opinion as to the mechanism of their production.

It seems to be allowed on all sides that the consequences in question owe their origin to the presence of pus in the blood; but how the pus gets or is produced there is variously accounted for. It has been attributed, 1st, to the absorption of pus from the wound by the capillaries and veins open in it; 2d, to phlebitis; 3d, to metastasis; 4th, to the purulent diathesis or pyœmia; 5th, to the evolution of preexisting tubercles.

M. d'Arcet, without discussing the merits of these different theories, and without adopting any opinion as to the mode by which the pus is mixed with the blood, assumes this ultimate result of all these views to be established, and then proceeds to describe the consequences of various experiments which he made upon animals by injections of this fluid in different states into their veins. Previous to doing so he remarks that it has sometimes been asserted that multiplied abscesses, as he designates them, or purulent deposits, as we usually call them, have this peculiarity, that in the organ or part where they appear, none of the other ordinary evidences of inflammation are present, but that the pus seems as if deposited without any previous process of the kind. This he declares to be an error, for that he has always found a red spot of inflammation to precede their appearance, and when pus is actually present, coagulable lymph is deposited around it, which, becoming organized, assumes the appearance of a sort of cyst, having its internal surface as flocculent as that of the chorion itself.

If we take healthy pus, such as is the product of phlegmonous inflammation, and expose it to the action of oxygen gas, directly or through the intestines of a living animal, or a piece of gold-beater's leaf, it soon absorbs a volume and a half of the gas, producing only a fifth of carbonic acid. While this takes place the pus-globules run together and agglomerate, and form an amorphous layer or coat, which floats on the subjacent liquid. If exposure to the air or oxygen be continued, the whole becomes offensive and putrid, without, however, the layer above mentioned becoming redissolved; and if we separate it by means of the filter we obtain a yellowish-green fluid, blackening silver, and evidently containing sulphuretted compounds, upon which a portion, yet not all of the poisonous properties depend, seeing that these remain when the former have been removed by mixing litharge with the fluid.

Taking the *first product* of the spontaneous decomposition of pus, the amorphous, inert, and insoluble layer or substance, and having repeatedly washed it, M. d'Arcet injected it into the jugular veins of rabbits and dogs. Where the animals were not immediately killed by the injection, which frequently happened, sometimes they got up, after a few moments of syncope, remained for a longer or shorter time in a state of prostration and weakness, and then recovered, without any traces of the experiment being left; at other times the depression increased, the animal retired into a corner, the pulse got frequent and hard, the respiration hurried, and death ensued, never later than forty hours after the experiment—death taking place quietly, without diarrhœa, vomiting, &c. On examination there were found phlyctenæ of the lungs, subpleural ecchymoses, penetrating into the parenchyma, with a nodule of well-marked hepatization in their centre. In two instances only, one in a dog at the end of forty-eight hours, (the time of death of the other was not noted,) where the lungs were covered with ecchymoses, in several of them were found a circumscribed purulent deposit, (noyau purulent circonscrit,) identical with those met with in man. In both cases there was likewise effusion of serum into the cavity of the pleura.

In none of these instances, however, were any other effects or symptoms produced than those dependent on the local inflammation, i. e. of the lungs. There were none of the terrible symptoms which indicate a more profound affection of the system—there was disease but no diathesis.

These effects, then, correspond to those produced by the injection of quicksilver by Cruveilhier and Gaspard, of charcoal in powder by Magendie, of cerebral substance by Dupuy, of the blood of the slug by Gaspard, of particles of gold by D'Arcet himself. And he therefore attributes them to a common cause, the insoluble, amorphous, and pulverulent nature of the substances injected rendering them incapable of elimination, and, from their size, of circulating in the capillaries. All these substances, when injected, produced no other results than local lobular inflammation of various organs, without other symptoms than those of pure phlegmonous inflammation.

The effects of the injection of the *second product* of the spontaneous decomposition of pus, of the yellowish-green putrid fluid, freed from all insoluble matters, were very different. Hiccup, vomiting, diarrhœa, rigors, fever, dyspnœa, opened the scene, followed by the most marked adynamic state, depression, stupor, involuntary evacuation of urine and fæces, pale appearance of the mucous membrane, different hemorrhages, abdominal pains, and the most complete prostration, and death in five hours. On dissection, the lungs were found of a violet colour, infiltrated and indurated, as in œdema, their surface covered with small spots of sub-pleural and inter-lobular ecchymosis. Similar ecchymoses existed on the spleen, liver, and intestines; the inner membrane of the aorta was reddened; the blood fluid, black, greenish, containing grumous portions, which broke down under the fingers, without communicating the sensation of fibrin. These alterations, our author remarks, display a general malady, one of the whole system, one by which the life of all the organs is destroyed, instead of death from the interruption of a single function. It is no longer a disease but a diathesis—the liquid injected acting like a leaven has communicated its poisonous and deleterious properties to the entire blood.

If we inject *healthy pus*, before it has undergone any decomposition from exposure to the air or oxygen, we by no means so frequently produce purulent deposits, as has been by some asserted. Out of eleven or twelve experiments, M. D'Arcet only twice succeeded in obtaining this result; in the majority of instances the putrid symptoms alone supervened.

Supposing that this failure might depend on human pus having been injected, he put setons into the dog and rabbit, and injected the pus so obtained, but without any difference in the effects.

With the results of these experiments upon animals before him, M. D'Arcet then proceeds to draw a parallel between them and those observed in man, and having established their entire correspondence, comes to the conclusion that the disease called *purulent absorption*, *phlebitis*, *purulent infection*, *purulent diathesis*, is a complex malady, where two very distinct classes of phenomena are observable, but which occur so combined, that hitherto they have been confounded together. These phenomena are:

1. A disease of the respiratory, hepatic, or other organs; a local inflammation, dependent on a mechanical cause, the capillary tissue being embarrassed by insoluble or pulverulent principles developed in the pus by its exposure to the oxygen of the air (in the lungs), and not producing other constitutional effects than those of phlegmonous inflammations of the same organs.

2. A miasmatic poisoning, caused by the absorption and circulation of some principles of the pus itself become putrid, acting on the blood in a special manner, and producing grave general symptoms, especially characterized as adynamic, and such as indicate a class of diseases, where the entire organization is intimately deranged, as the plague, typhus fever, purpura, glanders, &c.

The explanation given of the origin and mechanism of these two effects is this. Purulent matter, being mixed and circulating with the blood, reaches the lungs, and being there exposed to the action of oxygen, undergoes such changes as would happen to it, as an unorganized substance, out of the body. Its elements separate into two parts, the globules absorbing oxygen, increase in size by their reunion, and become incapable of traversing the capillaries, the caliber of which they obstruct, in the same way as mercury, gold, or charcoal, and thus produce those series of phenomena which result from those substances being introduced into the circulation. The liquid part, under the same influence, acquires putrid properties, which produce the effects above described, effects absolutely identical with those occurring from the simultaneous circulation of blood and putrid matter. Having given this explanation, our author adds, that he thinks it is impossible to admit that pus, in substance, can be absorbed by the capillaries; the laws of endosmosis applying, as they do, only to soluble substances, do not allow this. But although the passage of pus itself does not take place, that of the purulent serosity, deprived of its globules, readily does so, and hence, in addition to inoculation strictly so called, there is another road opened for putridity entering the system.

The disappearance, by absorption, of collections of matter, or abscesses, without any remarkable disturbance of the system, is next alluded to. M. D'Arcet allows the fact, and endeavours to account for it. Having observed in several cases where purulent deposits had existed, as ascertained by dissection, that during life the urine was albuminous, he was led to infer that in cases where abscesses dispersed without bad consequences, this might also be the case; and he relates two instances (in man) in which he had an opportunity of observing and ascertaining that whilst the tumours were dispersing the urine became albuminous. But although this accounts for the serous portion of pus with its albumen, which indeed forms by far the largest part of it, what becomes of the pus-globules? Referring to the observations of Dupuytren, we believe that these remain in the part, and constitute the greasy, putty-like substance met with by that distinguished surgeon and others, in the seat of chronic abscesses which had spontaneously disappeared.

---

